# Optimizing Performance of Coaxis Planar-Gated ZnO Nanowire Field-Emitter Arrays by Tuning Pixel Density

**DOI:** 10.3390/nano12050870

**Published:** 2022-03-05

**Authors:** Songyou Zhang, Xiuqing Cao, Guofu Zhang, Shaozhi Deng, Jun Chen

**Affiliations:** State Key Laboratory of Optoelectronic Materials and Technologies, Guangdong Province Key Laboratory of Display Material and Technology, School of Electronics and Information Technology, Sun Yat-Sen University, Guangzhou 510275, China; zhangsy35@mail2.sysu.edu.cn (S.Z.); caoxq7@mail2.sysu.edu.cn (X.C.); zhanggfu@mail.sysu.edu.cn (G.Z.); stsdsz@mail.sysu.edu.cn (S.D.)

**Keywords:** ZnO nanowire, gated field emitter arrays, electrical contact, transconductance

## Abstract

Gated ZnO nanowire field emitter arrays (FEAs) have important applications in large-area vacuum microelectronic devices such as flat panel X-ray sources and photodetectors. As the application requires high-pixel-density FEAs, how the pixel density affects the emission performance of the gated ZnO nanowire FEAs needs investigating. In this paper, the performance of coaxis planar -gated ZnO nanowire FEAs was simulated under different pixel sizes while keeping the lateral geometric parameter in proportion. The variations in emission current and gate modulation with pixel size were obtained. Using the obtained device parameters, the coaxis planar-gated ZnO nanowire FEAs were prepared. Field emission measurement results showed that a current density of 3.2 mA/cm^2^ was achieved from the fabricated ZnO nanowire FEAs when the gate voltage was 140 V. A transconductance of 253 nS was obtained, indicating effective gate control. The improved performance is attributed to optimized gate modulation.

## 1. Introduction

Vacuum microelectronic devices, such as X-ray sources [1,2,3], photodetectors [4,5] and parallel electron beam lithography [6], impose high requirements on large-area field emitter arrays (FEAs). One-dimensional (1D) materials are considered ideal candidates for large-area FEAs due to their high aspect ratio and unique properties [7,8,9,10,11]. In particular, ZnO nanowires have the advantages of large-area uniform preparation, low cost and good compatibility with microfabrication techniques, and thus extensive studies have been carried out on the field emission properties of ZnO nanowires [12,13,14,15,16,17]. Gated ZnO nanowire FEAs have been fabricated and proven to be capable of emission current modulation and addressability [18,19,20,21]. The feasibility of using ZnO nanowire FEAs in large-area vacuum microelectronic devices has also been verified [22].

Various gated structures have been proposed for achieving gated ZnO FEAs, including normal-gate, under-gate and planar-gate [18,19,20,21,23,24,25,26,27,28]. Li et al. reported fork-finger-shaped planar-gated ZnO nanowire FEAs, and a field emission display using the FEAs displayed a cartoon of a running dog and Chinese characters in full screen [27]. Zhao et al. reported comb-shape gated ZnO nanowire FEAs [18]. By the combination of under-gate and planar-gate, the structure could avoid the effect of the etching process on the cathode electrode and maintain the field emission properties of the nanowires. Planar-gate ZnO nanowire FEAs using a ring-shaped cathode were proposed in the work of Zhao et al. [19]. The ring-shaped ZnO nanowire patterns increase the edge area of the cathode, suppress the diode effect and thus exhibit excellent electron emission characteristics. Liu et al. reported ZnO nanowire FEAs with coaxis planar-gate electrodes and focusing electrodes [21], which demonstrated the capabilities of line addressing and focusing. Cao et al. further proposed coaxis planar-gated ZnO nanowire FEAs with an in-plane focusing gate electrode and investigated their addressing and focusing characteristics [20]. Cao et al. also used gated ZnO nanowire FEAs to develop a fully vacuum-sealed addressable flat-panel X-ray source array device, which achieved addressable X-ray emission with gate modulation [3]. The available experimental results show that the coaxis planar-gated FEAs have the advantages of simple structure and easy integration with ZnO nanowires, which are promising for large-area vacuum microelectronic device applications.

To further meet the requirements of high-resolution device applications, one has to increase the pixel density of the FEA devices. As the number of pixels per unit area increases, the size of each pixel decreases accordingly. In the planar-gated ZnO nanowire FEAs, the emission from nanowires is regulated by both the anode-induced electric field and the gate-induced electric field. When scaling down the pixel size, the size of the gate electrode and the distance between the gate and cathode change. Accordingly, the strength and direction of the gate-induced electric field at the tip of the nanowires change. In the meantime, the number of nanowires in each pixel also changes when the pixel size scales down, which will affect the effective number of nanowire emitters. Therefore, it is interesting to investigate how the performance of gated nanowire FEAs varies with the pixel density. However, this issue has not been addressed yet.

In this paper, the gate modulation of the electrical field and emission current of coaxis planar-gated ZnO nanowire FEAs with different pixel sizes is simulated using a two-dimensional (2D) model. Then, geometric sizes of gated ZnO nanowire FEAs were designed according to the simulation results and the devices were prepared. The field emission characteristics of the prepared FEAs were measured. This work has significance for improving the resolution and performance of large-area ZnO nanowire FEAs and realizing their applications.

## 2. Device Structure and Simulation

**Figure 1:**
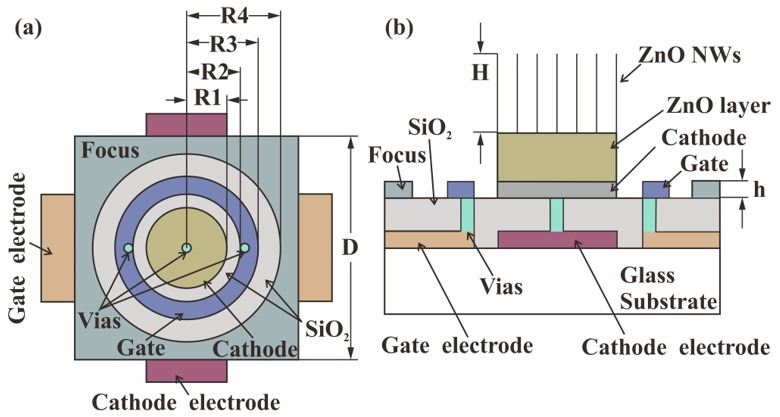
Schematic of the coaxis planar-gated ZnO nanowire FEAs with an in-plane focusing gate electrode. (**a**) Top view of one pixel; (**b**) cross-sectional view of one pixel.

The structure of the coaxis planar-gated ZnO nanowire FEAs with an in-plane focusing gate electrode is shown in Figure 1. In this structure, the cathode, gate and focusing electrode are arranged from inside to outside on the same top-layer plane. The bottom electrodes of the cathode and gate are perpendicularly arranged to achieve addressable pixels. The cathode and the gate at the top layer are connected to the corresponding electrodes at the bottom layer through via holes (Vias) [20]. The ring-shaped gate electrode can control the electron emission of electrons from ZnO nanowires. The focus electrode is located outside the gate electrode, and the trajectory of the emitted electrons can be controlled when a voltage is applied.

**Figure 2:**
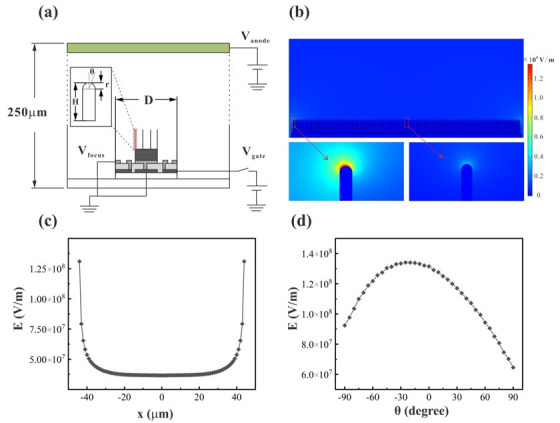
(**a**) Schematic of the model for simulating the electric field; (**b**) a typical simulation result with D = 250 µm, R1 = 45 µm, R2 = 60 µm, R3 = 80 µm, R4 = 105 µm, H = 4 µm, r = 25 nm, h = 0.3 µm, *V_gate_* = 150 V; (**c**) electric field at the tip of nanowires in a pixel obtained from (**b**); (**d**) typical electric field at different angles of the nanowire tip.

A simulation was carried out using Multiphysics software (COMSOL 5.3a, Stockholm, Sweden) based on a 2D model to investigate the electric field of ZnO nanowire FEAs when varying the pixel densities. The parameters of the device were defined as shown in Figure 1a,b. Each pixel occupies a square area with a side length of D. R1 is the radius of the cathode electrode, R2 and R3 are the inner and outer radii of the ring gate, R4 is the inner radius of the focus electrode, and h is the thickness of these electrodes. The nanowires are modelled as cylinders with a hemispherical tip, and the distance between the nanowires is 1 micron, according to the actual grown nanowires. The height of the nanowire is defined as H and the diameter of the nanowire is fixed at 50 nanometers. θ is the angle between a point on the tip of the nanowire and the central axis of the nanowire. The nanowires are located on the cathode electrode. Usually, a layer of ZnO film exists underneath the nanowires due to the preparation method of the nanowires. The thickness of the ZnO layer is fixed (1 μm). Then, we used the 2D model in the simulation of the electric field, as shown in Figure 2a. The distance between anode and cathode was set to 0.25 mm. Anode voltage (*V_anode_*) was set to 1500 V, while the cathode and the focus electrodes were grounded. The anode voltage value was chosen to avoid the diode emission effect induced by the anode. When the anode voltage is too high, the electric field at the tip of the nanowire is high even when the gate voltage is very low. This will lead to significant diode emission. Furthermore, when the anode-induced electric field is dominant, the gate control of the emission current will be low.

In the simulation, we adjusted the lateral parameters of the device and the height of the nanowires, while keeping the proportion of the R1, R2, R3 and R4. The details of the parameters for simulation are shown in Table 1. The side length D varied from 250 μm to 25 μm. The height of nanowires was chosen to be 3, 4 and 5 microns. Moreover, *V_gate_*, the voltage applied to the gate electrode, was chosen to be 150 V, 130 V and 110 V when the pixel size was changed. Figure 2b gives an example of a typical simulation result. 

From the simulation, the maximum electric field (*E*) at the tip of the nanowires could be obtained, as shown in Figure 2c. Due to the shielding effect between the nanowires, the electric field at the tip of the outermost nanowire is much higher than those of the inside nanowires. The field emission current density (*J*) follows the Fowler–Nordheim (F–N) equation [29,30]:

(1) $$J=A\frac{\beta^{2}E^{2}}{\phi^{2}}\exp(-B\frac{\phi^{3/2}}{\beta E}),$$

where *E* is the electric field, *ϕ* is the work function, and *A* and *B* are the F–N constants. From the F–N equation, we found that the emission current density at the tips of the outermost nanowires is much higher than those of the inside nanowires. Thus, the emission current can be approximately assumed to originate from these outmost nanowires. Figure 2d shows a typical electric field distribution at the tip of the outermost nanowire. We found that the electric field distribution at the tip of the nanowires is asymmetrical, i.e., the maximum electric field is not at point θ = 0°. This is because the position of the outermost nanowire is asymmetrical with respect to the ring gate electrode.

In order to calculate the emission current *I_nanowire_* from the outermost nanowire, we divided the tip into 36 segments. The field emission current density (*J_segment_*) for each segment was then calculated using the F–N equation. Then, the current for each segment was approximated as the current density (*J_segment_*) multiplied by the corresponding area (*S_segment_*), and *I_nanowire_* is the sum of the current on each segment, i.e.,

(2) $$I_{nanowire}=\sum_{i=1}^{36}(J_{segment}\cdot S_{segment}).$$

We calculated the emitted current (*I_pixel_*) of one pixel using the following equation:

(3) $$I_{pixel}=I_{nanowire}\cdot N,$$

where *N* is the number of outermost nanowires on the cathode pattern. The current of the device (*I_device_*) was approximated as *I_pixel_* multiplied by the number of pixels in the device. In addition, the transconductance (*g_m_*) could describe the effect of gate control, which is determined using

(4) $$g_{m}={\left.\frac{dI}{dV_{gate}}\right|}_{V_{anode}=const.},$$

where *V_gate_* is the gate voltage and *V_anode_* is the anode voltage, while *I* is the emission current. We used the current of one pixel (*I_pixel_*) to calculate the transconductance of one pixel (*g_m-pixel_).*

**Figure 3:**
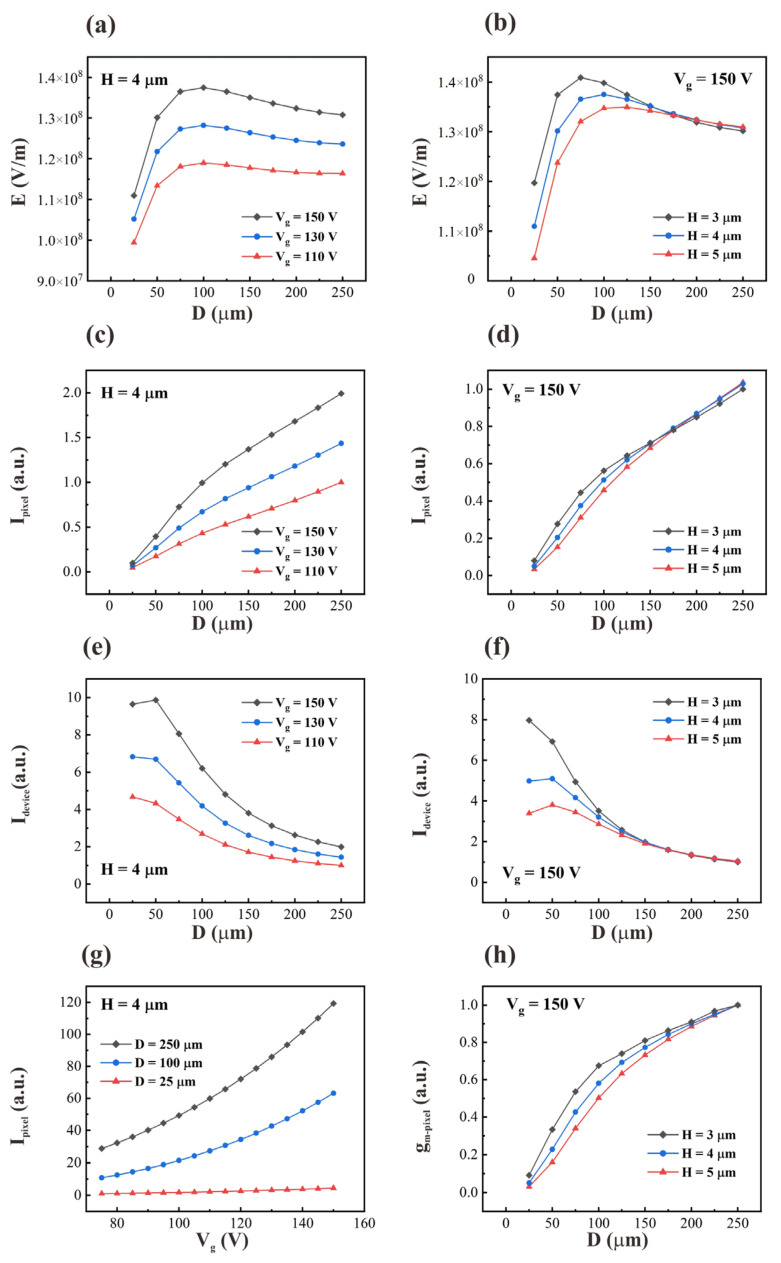
The simulation results of the variations of the electric field strength with pixel size for (**a**) different gate voltages (H = 4 μm) and (**b**) different nanowire heights (Vg = 150 V); variations in one pixel current with pixel size for (**c**) different gate voltages (H = 4 μm) and (**d**) different nanowire heights (Vg = 150 V); variations in device current with pixel size for (**e**) different gate voltages (H = 4 μm) and (**f**) different nanowire heights (Vg = 150 V); (**g**) variations in one pixel current with gate voltages for different pixel sizes (H = 4 μm); (**h**) variations in one pixel transconductance with pixel size for different nanowire heights (Vg = 150 V).

The results of the simulation under different device parameters are shown in Figure 3. The simulation results of the maximum electric field at the tip of the outermost nanowires are presented in Figure 3a,b. Figure 3a shows the relationship between the electric field and pixel size for a fixed nanowire length (4 µm) and different gate voltages. Figure 3b shows the relationship between electric field and pixel size for different nanowire lengths under a fixed gate voltage (150 V). The simulation results show that the electric field strength increases first and then decreases as the pixel size decreases. Moreover, as the nanowires become longer, the pixel size corresponding to the maximum electric field strength becomes larger. The highest electric field strength occurs at pixel sizes of 75, 100 and 125 µm for nanowire lengths of 3, 4 and 5 µm, respectively. Furthermore, the smaller the pixel size, the more the electric field strength is influenced by the length of the nanowires. After the pixel size exceeds 150 µm, the electric field strength is almost independent of the nanowire length.

Figure 3c,d give simulation results for the current of one pixel. They show that the pixel current decreases as the pixel size decreases, and the pixel current decreases more quickly when the pixel size is smaller than 100 µm. Figure 3e,f give the simulation results for the total emission current of the device. They show that the total current of the device increases as the pixel size decreases. This means that when the electric field starts to decrease as D < 100 μm, the total current of the device still increases. It is worth noting that when the pixel size is smaller than 50 µm, the device current tends to be unchanged or even decreases. This phenomenon becomes more pronounced at higher gate voltages and nanowire lengths. Figure 3g gives the simulation results of the pixel current under the range of gate voltage commonly applied in practical devices. The pixel current increases with increasing gate voltage and increases more rapidly when the gate voltage increases. Figure 3h shows the simulation results of the transconductance of one pixel under different pixel sizes when the gate voltage is 150 V. The results show that the transconductance of one pixel decreases gradually as the pixel size decreases and decreases more rapidly when the pixel size is smaller than 100 µm. 

**Figure 4:**
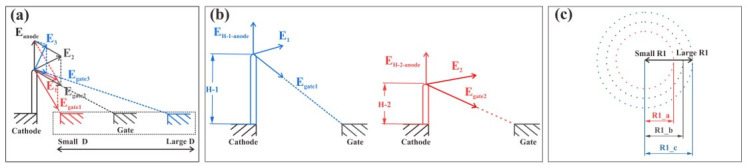
Schematic illustrations used to explain the simulation results. (**a**) The gate-induced electric field when the pixel size changes; (**b**) the electric fields under two different nanowire lengths; (**c**) the number of nanowires when R1 changes. The dot represents the outermost nanowires located at the periphery of the cathode.

The simulation results can be interpreted using the schematic illustrations shown in Figure 4. Figure 4a shows the gate-induced electric field when the pixel size changes. As the size of the pixel changes, the distance between the gate and the cathode changes. As shown in Figure 4a, although the gate-induced electric field becomes larger as the distance between the gate and cathode becomes smaller, the direction of the gate-induced electric field also changes. When D is large, the superimposed electric field (E) of the gate-induced electric field (*E_gate_*) and the anode-induced electric field (*E_anode_*) increases when D decreases. However, when D decreases, the upward electrical field component in the gate-induced electric field becomes smaller. Therefore, there is a D at which the superimposed electric field and the current density calculated from the electric field have a maximum value (as shown in Figure 3a).

Figure 4b illustrates the electric fields under two different nanowire lengths, which can explain the effect of the length of the nanowires on the gate control on the electric field at their tips. The nanowires with higher length are relatively farther away from the gate, resulting in a smaller electric field. Meanwhile, when the pixel size is large, the nanowire length is much smaller than the pixel size and therefore the effect of the nanowire length on the electric filed is minimal, which explains why the variation in nanowire length has little effect on the electric field when the pixel size is larger than 150 µm (as shown in Figure 3b). However, longer lengths of nanowires have a higher field enhancement factor and will promote the emission of electrons.

Figure 4c illustrates the number of nanowires when the radius of the cathode (R1) changes, which can explain the variation in the field emission current with pixel density. When R1 becomes smaller, the number of outermost nanowires involved in field emission decreases, leading to a lower emission current in a single pixel. This explains why the current on one pixel is reduced (as shown in Figure 3c,d). Figure 3c,d also show that the current density decreased more dramatically when D was smaller than 100 µm. This is because the electrical field decreases when the pixel size decreases to less than 100 µm.

For the device current, as the pixel size deceases, the number of pixels increases and the number of nanowires per pixel decreases. The result of the combined action determines the overall device current, and a maximum occurs at D = 50 µm (as shown in Figure 3e,f). 

In summary, as the pixel size decreases, the field emission performance of the device improves within a certain range due to changes in the distance between electrodes and the number of pixels. The simulation results provide guidance for improving the field emission performance by adjusting the pixel size. An appropriate reduction in pixel size will balance the control capability of the gate electrode and field emission current.

## 3. Device Fabrication

Based on the above simulation results, the coaxis-gated ZnO nanowire FEA device was designed with 200 × 200 arrays of patterned ZnO nanowire field emitters, and the size of each pixel was 100 μm × 100 μm. This pixel size was chosen as a compromise between the current of the device and the transconductance of one pixel. For each pixel, the cathode pattern had a radius of 18 μm and was surrounded by a ring electrode acting as the control gate. The inner and outer diameters of the ring-shaped gate electrode were 24 and 32 μm, respectively.

**Figure 5:**
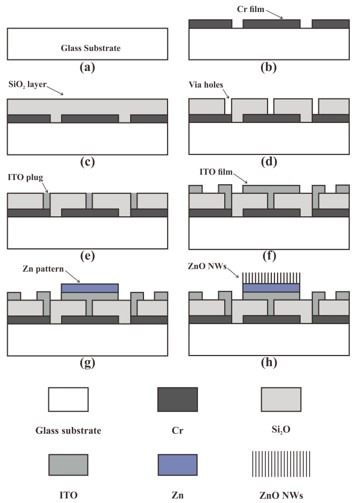
Schematic diagram of the preparation process of ZnO nanowire FEAs. (**a**) Substrate cleaning; (**b**) Cr layer deposition; (**c**) SiO_2_ layer deposition; (**d**) via holes etching; (**e**) deposition of ITO plug; (**f**) ITO film deposition; (**g**) Zn film deposition; (**h**) growth of ZnO nanowires.

A four-step-mask process combining the microfabrication process and thermal oxidation technique was used to prepare the coaxis-gated ZnO nanowire FEAs on glass substrates. Figure 5 gives a brief description of the fabrication process. First, the glass substrate was ultrasonically cleaned with acetone, ethanol and deionized water. A 120-nm-thick chromium layer was deposited on the glass substrate by magnetron sputtering, and the patterned bottom electrode was obtained by a lift-off process (Figure 5b). Then, a layer of 1.5-μm-thick silicon oxide film was deposited by plasma-enhanced chemical vapor deposition (PECVD) as the insulating layer between the bottom electrode and the upper electrode (Figure 5c). After this, reactive ion etching (RIE) was used to etch the via holes (Figure 5d). The via holes were filled with a indium tin oxide (ITO) plug to connect the upper and bottom electrodes (Figure 5e). Then, the patterns of the upper cathode, gate and focus electrode were obtained through photolithography, and a 320-nm-thick indium tin oxide (ITO) thin film was deposited by direct-current (DC) magnetron sputtering (Figure 5f). Later, the Zn film was deposited by electron beam evaporation deposition and the Zn pattern was obtained by a lift-off process (Figure 5g). Finally, the ZnO nanowires were synthesized by thermal oxidation in air at 470 °C for 3 h (Figure 5h). In our experiment, the ITO film was annealed simultaneously during the thermal oxidation process. An ITO electrode with good conductivity could be obtained after the thermal oxidation process. It is worth noting that, compared with previous devices [20,21], ITO plugs for vias were adopted in this study, which could improve the reliability of the electrical contact between the upper and bottom electrodes.

## 4. Device Characterization

**Figure 6:**
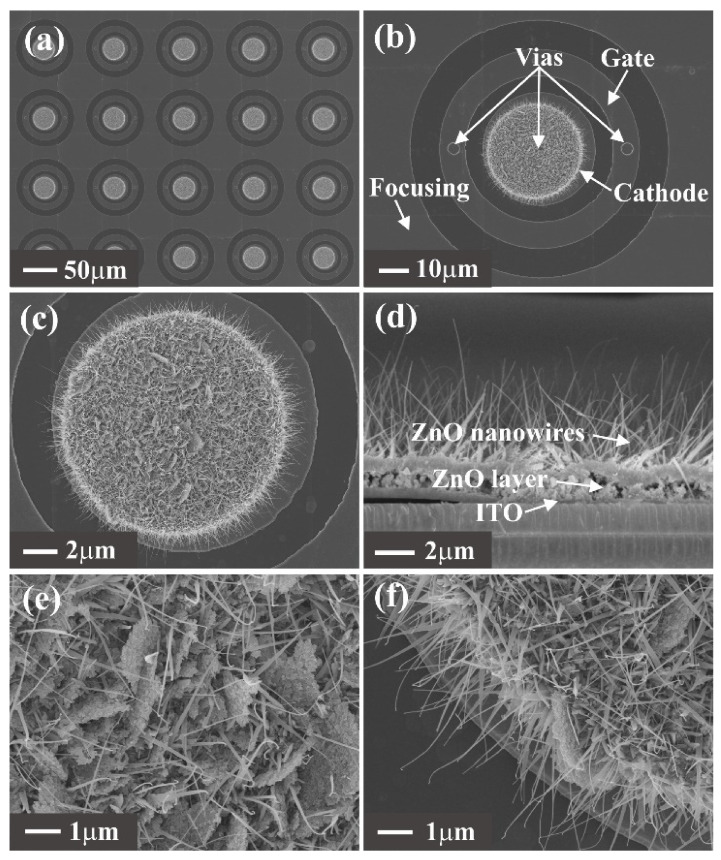
SEM images of the fabricated ZnO nanowire FEAs. (**a**) ZnO nanowire FEAs; (**b**) single pixel of ZnO nanowire FEAs; (**c**) the cathode pattern; (**d**) cross-sectional view of the ZnO nanowires; (**e**) ZnO nanowires at the inner part of the cathode pattern; (**f**) ZnO nanowires at the edge of the cathode pattern.

The fabricated ZnO nanowire FEAs were observed by a scanning electron microscope (SEM; Zeiss SUPRA 60, Jena, Germany), and the images are presented in Figure 6. Figure 6a shows the top-view SEM image of the ZnO nanowire array, where each pixel in the array is uniformly distributed. Figure 6b shows the morphology of a single pixel. We can see the control gate electrode and focusing electrodes in the same plane and the two via holes on the control gate electrode. The morphology of ZnO nanowire FEAs shows that the prepared device is consistent with our design. Figure 6c shows an enlarged SEM image of the cathode pattern in a pixel. The cathode pattern is covered with dense ZnO nanowires. Figure 6d shows a cross-sectional view of a pixel, clearly showing the ITO film, ZnO layer and ZnO nanowires. It indicates that the ZnO nanowires are grown nearly vertically and uniformly on the ZnO layer. Figure 6e,f show the ZnO nanowire within and at the edge of the cathode pattern. The average height and diameter of ZnO nanowires are in the ranges of 3–5 μm and 10–30 nm, respectively, which is similar to previous reports [3,19,20,21,31].

The ZnO nanowire FEA device was measured in a vacuum chamber with a base pressure of ∼2 × 10^−5^ Pa. We used green phosphor as an anode and the distance between anode and cathode was 0.25 mm. The anode voltage was biased at 1.2 kV, the cathode electrodes and focusing electrodes were grounded, and the gate voltage was increased from 70 to 120 V. The field emission current–voltage characteristics were recorded with Keithley 2450 and 2657A source meters. In addition, the field emission images were recorded using a digital camera.

**Figure 7:**
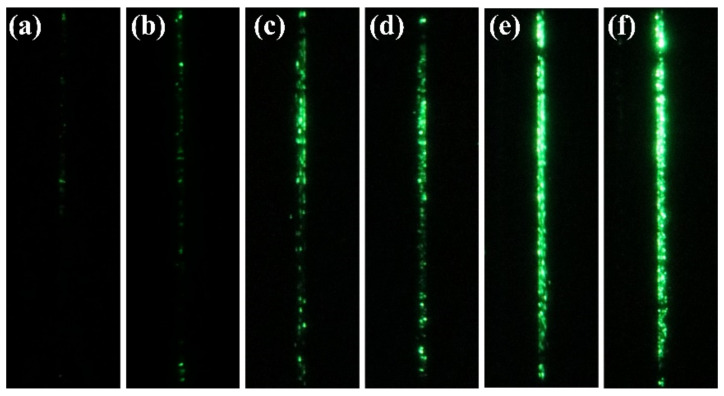
Emission images of two columns (200 × 2) of the arrays driven with different gate voltages. (**a**) Vg = 70 V; (**b**) Vg = 80 V; (**c**) Vg = 90 V; (**d**) Vg = 100 V; (**e**) Vg = 110 V; (**f**) Vg = 120 V.

Figure 7 shows the field emission images acquired under different gate voltages. The emission results indicated that the gate voltage can successfully modulate the emission current. It can be seen from the fluorescent screen that there was not enough electron emission when only 1.2 kV anode voltage was applied. As the gate voltage increased gradually, obvious emission could be observed.

**Figure 8:**
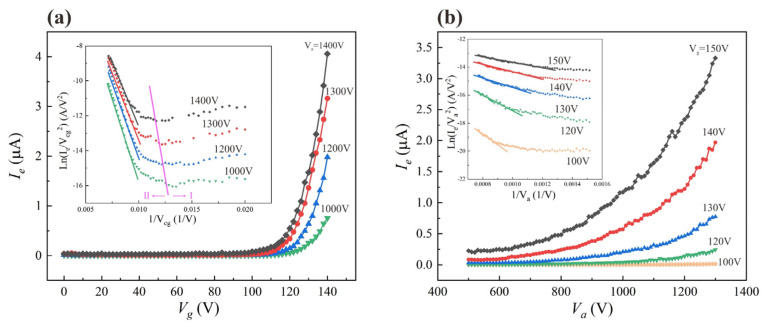
(**a**) Curves of field emission current and gate voltage (I_e_−V_g_) at different V_a_ values; (**b**) curves of field emission current and anode voltage (I_e_−V_a_) at different V_g_ values. The inset shows the corresponding F–N plots.

We measured two columns (200 × 2) of the arrays in the device under different control gate voltages (V_g_) and anode voltages (V_a_), while other unselected control gate lines and focus gate electrodes were connected to the ground. The control gate voltage varied from 0 to 140 V and the anode voltage varied from 500 to 1300 V. Figure 8 shows the emission current results collected at the anode (I_e_). Figure 8a gives the I_e_−V_g_ plots obtained under various anode voltages, and Figure 8b gives the I_e_−V_a_ plots obtained under various gated electrode voltages. The insets show the corresponding F–N plots. Both the I_e_−V_g_ plots and the I_e_−V_a_ plots confirmed the modulation function of the control gate. According to Figure 8a, it is observed that when the gate voltage rises to 100 V, the anode current then begins to increase significantly, and the value reaches up to 4.06 μA when the gate voltage increases to 140 V under V_a_ of 1400 V. The emission currents at V_g_ = 140 V and V_a_ of 1300, 1200 and 1000 V were 3.16, 1.98 and 0.75 μA, respectively. The emission current varies greatly at different anode voltages, even though the gate voltages are the same. We believe that this might be due to the fact that the field emission is induced by the combination action of both the anode and control gate voltage. Moreover, the emission currents are shown in Figure 8b for when V_a_ varied from 500 to 1300 V and V_g_ was 100, 120, 130, 140 and 150 V, respectively. The results show that the emission current increases more rapidly with the anode voltage at high V_g_ than those at low V_g_. This also indicates that the emission current was affected by both V_a_ and V_g_, which is consistent with the result in Figure 8a. The electric field at the tip of the ZnO nanowire was induced by both the anode voltage and control gate voltage. When the V_g_ was low, the emission current was more sensitive to V_a_, and when V_g_ was high, the emission current was more sensitive to V_g_.

The F–N plots presented in the inset of Figure 8a consist of two regions, which is commonly observed in this type of gated FEA [20]. Region I is the region of low gate voltage and region II is the region of high gate voltage. In region I, the anode voltage is the main cause of electron emission, and the emission current induced by the control gate was very small. Therefore, positive slope curves that do not conform to the F–N equation are observed in region I. In region II, a typical F–N-type straight line with a negative slope is observed. This indicates that, in this region, the electron emission of the ZnO nanowire field emitter was induced by the control gate following the classical electron tunneling mechanism.

The emission current density, which was calculated by using the cathode emission area, was 3.19 mA/cm^2^, when V_g_ was 140 V and V_a_ was 1400 V. In addition, the transconductance of the device under this condition was obtained from Equation (4) as 253 nS.

Our work was compared with early ones. The structure, pixel density and emission characteristics of some planar gated ZnO nanowire FEAs are given in Table 2. The maximum emission current density obtained in this work is higher than the previously reported ZnO nanowire FEAs. The FEAs reported in this study have the highest resolution. The results verify the simulation that high-pixel-density FEAs facilitate the improvement of the emission current of the device.

## 5. Conclusions

A high-pixel-density coaxis planar-gated ZnO nanowire field emitter array has been designed and prepared. The device performance was simulated under different pixel sizes while keeping the lateral geometric parameter in proportion. Simulation results show that an appropriate reduction in pixel size could enhance the gate modulation capability and increase the current density of FEAs, which is due to the optimized cathode-to-gate distance and the direction of the gate-induced electric field. Coaxis planar-gated ZnO nanowire field emitter arrays with optimized parameters were fabricated. The fabricated device was successfully driven, and a high current density (3.2 mA/cm^2^) and transconductance (253 nS) were obtained. The results are significant for improving the performance of large-area ZnO nanowire FEAs for vacuum microelectronic device applications.

## Figures and Tables

**Table 1:** The details of the parameters for simulation.

D (μm)	R1 (μm)	R2 (μm)	R3 (μm)	R4 (μm)	H (μm)	r (nm)	h (μm)
250	45	60	80	105	3,4,5	50	0.3
…	…	…	…	…	…	…	…
25	4.5	6	8	10.5	3,4,5	50	0.3

**Table 2:** Comparison of the structure, pixel density and emission properties of gated ZnO nanowire FEAs reported in the previous literature.

Structure	Pixel Density (cm^−2^)	Maximum Field Emission Current Density (V_g_)	Ref
Coaxis planar-gated without focusing gate	493	44.21 μA/cm^2^ (90 V)	[19]
Coaxis planar-gated with in-plane focusing gated (line-addressing)	1564	1.21 mA/cm^2^ (150 V)	[21]
Coaxis planar-gated with in-plane focusing gate (pixel-addressing)	4000	1.99 mA/cm^2^ (120 V)	[20]
Coaxis planar-gated with high pixel density	10,000	3.19 mA/cm^2^ (140 V)	This work

## Data Availability

Data are contained within the article.
